# Molecular Epidemiology of Extended-Spectrum Beta-Lactamase and AmpC Producing *Enterobacteriaceae* among Sepsis Patients in Ethiopia: A Prospective Multicenter Study

**DOI:** 10.3390/antibiotics11020131

**Published:** 2022-01-19

**Authors:** Melese Hailu Legese, Daniel Asrat, Abraham Aseffa, Badrul Hasan, Adane Mihret, Göte Swedberg

**Affiliations:** 1 Department of Medical Laboratory Sciences, Addis Ababa University, Addis Ababa 1176, Ethiopia; 2 Armauer Hansen Research Institute, Addis Ababa 1005, Ethiopia; aseffaa@gmail.com (A.A.); amihret@gmail.com (A.M.); 3 Department of Medical Biochemistry and Microbiology, Uppsala University, 75108 Uppsala, Sweden; b.hasanvet@gmail.com (B.H.); Gote.Swedberg@imbim.uu.se (G.S.); 4 Department of Microbiology, Immunology and Parasitology, College of Health Sciences, Addis Ababa University, Addis Ababa 9086, Ethiopia; asratdan@gmail.com

**Keywords:** multicenter study, molecular epidemiology, ESBL, AmpC, *Enterobacteriaceae*, sepsis, Ethiopia

## Abstract

Extended-spectrum beta-lactamases (ESBLs) and AmpC producing *Enterobacteriaceae* are public health threats. This study aims to characterize ESBL and AmpC producing *Enterobacteriaceae* isolated from sepsis patients. A multicenter study was conducted at four hospitals located in central (Tikur Anbessa and Yekatit 12), southern (Hawassa) and northern (Dessie) parts of Ethiopia. Blood culture was performed among 1416 sepsis patients. *Enterobacteriaceae* (*n* = 301) were confirmed using MALDI-TOF and subjected for whole genome sequencing using the Illumina (HiSeq 2500) system. The overall genotypic frequencies of ESBL and AmpC producing *Enterobacteriaceae* were 75.5% and 14%, respectively. The detection of ESBL producing *Enterobacteriaceae* at Hawassa, Yekatit 12, Tikur Anbessa and Dessie was 95%, 90%, 82% and 55.8%, respectively. The detection frequency of *bla*_CTX-M_, *bla*_TEM_ and *bla*_SHV_ genes was 73%, 63% and 33%, respectively. The most frequently detected ESBL gene was *bla*_CTX-M-15_ (70.4%). The common AmpC genes were *bla*_ACT_ (*n* = 22) and *bla*_CMY_ (*n* = 13). Of *Enterobacteriaceae* that harbored AmpC (*n* = 42), 71% were ESBL co-producers. Both *bla*_TEM-1B_ (61.5%) and *bla*_SHV-187_ (27.6%) were the most frequently detected variants of *bla*_TEM_ and *bla*_SHV_, respectively. The molecular epidemiology of ESBL producing *Enterobacteriaceae* showed high frequencies and several variants of ESBL and AmpC genes. Good antimicrobial stewardship and standard bacteriological laboratory services are necessary for the effective treatment of ESBL producing *Enterobacteriaceae*.

## 1. Introduction

Globally, *Enterobacteriaceae* that harbor extended-spectrum beta-lactamase (ESBL) genes are spreading and causing serious infections, such as sepsis [1]. Sepsis is a life-threatening condition resulting from a dysregulated immune response to the infection, which ultimately results in organ dysfunction [2,3]. Antibiotic options for the management of septic patients caused by ESBL producing *Enterobacteriaceae* (ESBL-pE) is narrow, which can lead to longer hospital stays, increased hospital costs and increased mortality [4].

ESBL-pE has become a global health problem [5] because ESBL can make a diverse range of β-lactam antibiotics ineffective, including penicillins, cephalosporins and monobactams [6]. *Enterobacteriaceae* acquire and disseminate these ESBL-encoding genes horizontally, mainly through plasmids [7]. In addition to *Escherichia coli*, various species of *Klebsiella*, *Enterobacter*, *Serratia* and *Salmonella* are major ESBL producers in the family *Enterobacteriaceae* [8,9,10].

Generally, all beta-lactamase variants are classified into four classes, A (serine penicillinases), B (metallo-beta-lactamases), C (cephalosporinases) and D (oxacillinases), which give resistance to penicillins, most β-lactams, cephalosporins and cloxacillin, respectively [11]. Some key families of ESBL-encoding genes, such as *bla*_CTX-M_, *bla*_TEM_ and *bla*_SHV_ are grouped in class A [8,12,13]. The *bla*_CTX-M_ family, that spread rapidly during the last decade, originated from environmental bacteria and its variants have developed due to point mutations [14]. All *bla*_CTX-M_ variants are ESBL genes [8,13] in which *bla*_CTX-M-15_ is presently the most prevalent [10]. Other ESBL genes include *bla*_OXA_, *bla*_VEB_, *bla*_VER_ and *bla*_GES_ [7,8,13]. The co-presence of ESBL genes in the same species is a significant threat of transfer to other species [11].

*Enterobacteriaceae* that produce AmpC enzymes can also hydrolyze β-lactams and inhibitor–β-lactam combinations [15]. AmpC may be encoded in the chromosomes or plasmids of most members of *Enterobacteriaceae* [16]. Around the globe, several AmpC genes are detected in *Enterobacteriaceae* and of these genes the *bla*_CMY_ and *bla*_DHA_ families are the most common [17,18,19,20].

The World Health Organization (WHO) has listed ESBL producing *Enterobacteriaceae* as critical priority pathogens [21] and the magnitude of such strains is increasing worldwide [6,22,23]. However, there is scarcity of data related to the genetic epidemiology of ESBL and AmpC producing *Enterobacteriaceae* in sub-Saharan countries, including Ethiopia. It is a crucial time to determine the genetic epidemiology of ESBL and AmpC producing *Enterobacteriaceae* at a larger scale in order to guide future antimicrobial resistance control programs. Hence, this study aims to determine the molecular epidemiology of ESBL and AmpC producing *Enterobacteriaceae* among patients investigated for sepsis at four Ethiopian teaching/referral hospitals, which are located in the central, southern and northern parts of the country. These hospitals are serving millions of people in the surrounding catchment area and people who are referred to these hospitals.

## 2. Results

### 2.1. Sociodemographic Characteristics

In the present study, a total of 1416 patients investigated for sepsis from four different hospitals were enrolled. The number of patients from Tikur Anbessa Specialized Hospital (TASH) was 501, and the numbers from Yekatit 12 Specialized Hospital Medical College (Y12HMC), Dessie Referral Hospital (DRH) and Hawassa University Comprehensive Specialized Hospital (HUCSH) were 298, 301 and 316, respectively. The male participants were 55.3% while the females were 44.7%. The patients’ ages ranged from half a day to 90 years with a mean age of 8.85 years (Table 1).

### 2.2. Enterobacteriaceae: Frequencies and Distributions per Study Site

From the 1416 patients, blood cultures were performed at each study site, and a total of 301 *Enterobacteriaceae* isolated from all the study sites were subjected for whole genome sequencing (WGS). Of these, *Klebsiella pneumoniae* (*n* = 103), *Klebsiella variicola* (*n* = 74) and *Escherichia coli* (*n* = 53) were most frequently identified and their frequency varied between the 4 hospitals (Table 2).

### 2.3. Detection of bla_CTX__-M_, bla_TEM_ and bla_SHV_ Genes

**Figure 1:**
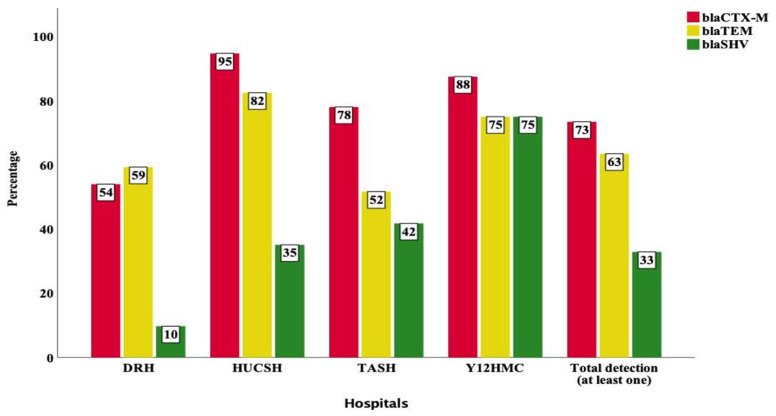
Frequency of *bla*_CTX-M_, *bla*_TEM_ and *bla*_SHV_ families detected at least once from *Enterobacteriaceae* subjected to WGS per study site. TASH—Tikur Anbessa Specialized Hospital; Y12HMC—Yekatit 12 Specialized Hospital Medical College; DRH—Dessie Referral Hospital and HUCSH—Hawassa University Comprehensive Specialized Hospital.

Among all *Enterobacteriaceae*, *bla*_CTX-M_, *bla*_TEM_ and *bla*_SHV_ were detected in 73%, 63% and 33% of the samples at least once (Figure 1). Among the *Enterobacteriaceae* isolated at each hospital, the detection of *bla*_CTX-M_ at HUCSH, Y12HMC, TASH and DRH was 95%, 88%, 78% and 54%, respectively. At DRH, *bla*_TEM_ (59%) was detected at a higher frequency while *bla*_SHV_ (10%) detection was low (Figure 1) At HUCSH, *bla*_TEM_ was detected at 82% while *bla*_SHV_ detection was relatively low (35%). At TASH, *bla*_TEM_ was more frequent than *bla*_SHV_. At Y12HMC, while the detection of *bla*_CTX-M_ was the highest with 88%, *bla*_TEM_ and *bla*_SHV_ detection was the same at 75%. Differences in the detection of *bla*_CTX-M_, *bla*_TEM_ and *bla*_SHV_ showed statistically significant associations per study site (*p* < 0.001).

**Figure 2:**
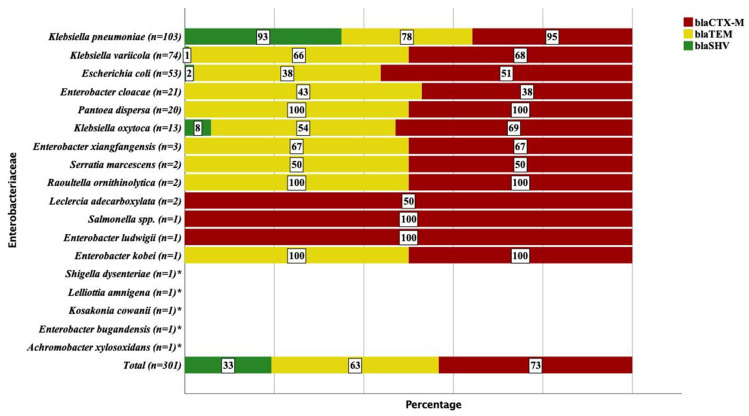
Frequency and distribution of *Enterobacteriaceae* that encoded *bla*_CTX-M_, *bla*_TEM_ and *bla*_SHV_ genes as detected using the whole genome sequence. * No *bla*_CTX-M_, *bla*_TEM_ and *bla*_SHV_ detected.

*K. pneumoniae* was the most frequent isolate and harbored high frequencies of *bla*_CTX-M_ (95%), *bla*_SHV_ (93%) and *bla*_TEM_ (78%) genes (Figure 2). Similarly, most *Enterobacteriaceae* were found to have *bla*_CTX-M_ and *bla*_TEM_ gene families with different detection rates between the strains. The detection of *bla*_SHV_ in *K. variicola* (1%) and *E. coli* (2%) was very low, while there was no detection of *bla*_SHV_ among *E. cloacae* (*n* = 21) and *P. dispersa* (*n* = 20). No *bla*_CTX-M_, *bla*_TEM_ and *bla*_SHV_ genes were detected in the rare isolates of *A. xylosoxidans*, *E. bugandensis*, *K. cowanii*, *L. amnigena* and *S. dysenteriae* (Figure 2).

### 2.4. Molecular Epidemiology of ESBL Producing Enterobacteriaceae

**Figure 3:**
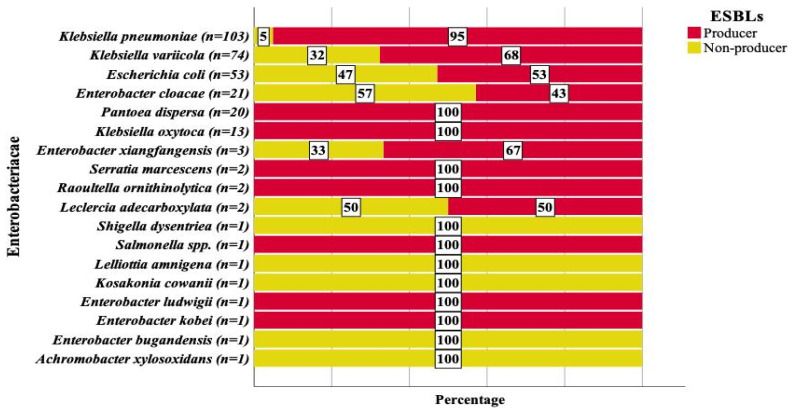
Frequency and distribution of *Enterobacteriaceae* that are ESBL and non-ESBL producers.

Of all the *Enterobacteriaceae* subjected to WGS, 75.5% encoded at least one ESBL gene. At least one ESBL gene was detected among 95% of *Klebsiella pneumoniae*, 68% of *K. variicola*, 53% of *E. coli and* 43% of *E. cloacae* (Figure 3). The frequencies of ESBL producing *Enterobacteriaceae* detected at HUCSH, Y12HMC, TASH and DRH were 95%, 90%, 82% and 55.8%, respectively (*p* < 0.001) (Table 3). Of the *Enterobacteriaceae* obtained from the neonatal intensive care units (*n* = 189) and pediatrics wards (*n* = 68), 73.5% and 87% had at least one ESBL gene, respectively (Table 3). Among the patients who showed blood culture positivity for *Enterobacteriaceae*, the possible risk factors for the increased ESBL producing *Enterobacteriaceae* were assessed. However, the multivariate analysis did not show any statistically significant association between the independent variables and higher frequencies of ESBL producing *Enterobacteriaceae* (Table 3). 

### 2.5. ESBL Genes

**Figure 4:**
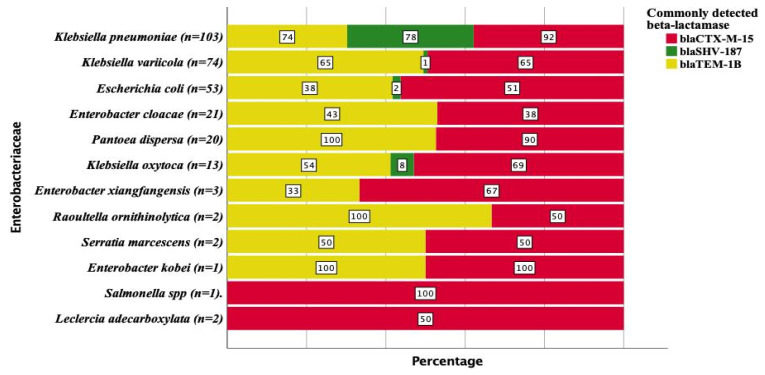
Frequency and distribution of *bla*_SHV-187_, *bla*_CTX-M-15_ and *bla*_TEM-1B_ that were detected frequently among *Enterobacteriaceae*.

Several variants of *bla*_CTX-M_ that are ESBL were detected among the *Enterobacteriaceae* sequenced from all the study sites (Table 4). The most frequent gene was *bla*_CTX-M-15_ with an overall detection rate of 70.4%. The frequency of *bla*_CTX-M-15_ at DRH, TASH, HUCSH and Y12HMC was 50.4%, 73.6%, 93% and 87.5%, respectively. In addition to *bla*_CTX-M-15_, two other *bla*_CTX-M_ variants were found at DRH only. *K. pneumoniae* (92%) was the most common *bla*_CTX-M-15_ producer (Figure 4). The detection of *bla*_CTX-M-15_ from *K. variicola, E. coli, P. dispersa* and *K. oxytoca* was 65%, 51%, 90% and 69%, respectively (Figure 4). *bla*_SHV-106_ was another ESBL gene detected at 3.7% frequency with a majority from DRH and TASH. *bla*_TEM-207_, *bla*_SRT-1_, *bla*_GES-11_ and *bla*_CMY-42_ were the other rare ESBL genes detected. *K. oxytoca* was found to carry several variants of the intrinsic *bla*_OXY_ gene, also classified as ESBL (Table 4).

### 2.6. Non-ESBL β-Lactamase Variants of bla_TEM_ and bla_SHV_

While some *bla*_TEM_ and *bla*_SHV_ are considered as common ESBL determinants, most variants detected in this study were different broad-spectrum beta-lactamase genes that were not ESBL (Table 5). The most frequently detected *bla*_TEM_ variant was *bla*_TEM-1B_ (61.5%) (Table 5) with frequencies of 57.5%, 80.7%, 49.5% and 72.5% at DRH, HUCSH, TASH and Y12HMC, respectively. While *bla*_TEM-1B_ and *bla*_TEM-1A_ were detected at all the four hospitals, other *bla*_TEM_ variants were mostly detected at DRH and TASH only. Other than the broad-spectrum beta-lactamases *bla*_TEM-1B_ and *bla*_TEM-1A_, no other *bla*_TEM_ variants were detected at Y12HMC. Of the five inhibitor-resistant broad-spectrum beta-lactamase TEM variants, three of them were detected at DRH. *K. pneumoniae, K. variicola, E. coli* and *E. cloacae* harbored *bla*_TEM-1B_ with the frequencies of 74%, 65%, 38% and 43% (Figure 4). 

Of the *bla*_SHV_ variants that are not ESBL, *bla*_SHV-187_ (27.6%) was the most frequently detected (Table 5) and most commonly at TASH (41.8%). Its detection at HUCSH and Y12HMC was 35% while it was more infrequent at DRH (9.7%). The *bla*_SHV-40_, *bla*_SHV-56_, *bla*_SHV-60_, *bla*_SHV-79_, *bla*_SHV-85_, *bla*_SHV-89_, *bla*_SHV-94_ and *bla*_SHV-172_ variants were only detected at Y12HMC. The common producers of *bla*_SHV-187_ were *K. pneumoniae* (78%), while *bla*_SHV-187_ was rare among *K. variicola* (1%), *E. coli* (2%) and *K. oxytoca* (8%) (Figure 4).

### 2.7. Co-Occurrence of Multiple ESBL Genes

In several *Enterobacteriaceae*, either the co-occurrence of multiple ESBL genes or one ESBL gene with other *bla*_TEM_ and *bla*_SHV_ variants was detected (Table 6). *Enterobacteriaceae* that carried the *bla*_CTX-M-15_ gene were found to harbor several other ESBL genes or non-ESBL variants of *bla*_TEM_ and *bla*_SHV_. The co-occurrence of *bla*_CTX-M-15_ and *bla*_TEM-1B_ (*n* = 166) was the most frequent gene combination followed by *bla*_CTX-M-15_ and *bla*_SHV-187_. While the 3 combinations of *bla*_CTX-M-15_ * *bla*_TEM-1B_ * *bla*_SHV-187_ were detected among 56 *Enterobacteriaceae,* the 4 *bla*_CTX-M-15_ * *bla*_TEM-1B_ * *bla*_SHV-187_* *bla*_SHV-106_ gene combination was detected from 9 *Enterobacteriaceae*. A combination of five multiple genes (*bla*_CTX-M-15_ * *bla*_TEM-1B_ * *bla*_SHV-187_ * *bla*_TEM-207_ * *bla*_SHV-106_) was detected from one *Enterobacteriaceae* (Table 6). 

### 2.8. Molecular Epidemiology of AmpC Producing Enterobacteriaceae

Among all the *Enterobacteriaceae*, 14% (*n* = 42/301) harbored at least one AmpC gene and seven of these were multiple AmpC gene carriers. *bla*_ACT_ (*n* = 22) was the most commonly detected AmpC gene family (Table 7). The variants of *bla*_ACT_ were *bla*_ACT-7_ (*n* = 9), *bla*_ACT-16_ (*n* = 6), *bla*_ACT-14_ (*n* = 3), *bla*_ACT-5_ (*n* = 2), *bla*_ACT-4_ (*n* = 1) and *bla*_ACT-15_ (*n* = 1). All these *bla*_ACT_ variants were found among *Enterobacter* species, and all were related to the intrinsic AmpC gene of this genus. Another related gene, *bla*_CMH-3_, was present in two samples of *E. cloacae* from DRH. Following *bla*_ACT_, *bla*_CMY_ (*n* = 13) was another commonly identified AmpC gene family. The different variants of *bla*_CMY_ were *bla*_CMY-6_ (*n* = 7), *bla*_CMY-148_ (*n* = 3), *bla*_CMY-2_ (*n* = 2) and *bla*_CMY-42_ (*n* = 1). Another AmpC gene detected was *bla*_DHA-1_ (*n* = 5). The two isolates of *S.* *marcescens* included also carried variants of the intrinsic *bla*_SRT_ genes. At least one acquired AmpC gene was identified from *K. pneumoniae* and *E. coli* (9% each) (Table 7). Of the *Enterobacteriaceae* that harbored AmpC (*n* = 42), 71% (*n* = 30/42) were found to co-produce ESBL genes while 29% (*n* = 12/42) were not ESBL producers.

Table 7 shows the frequency and distribution of the AmpC genes per hospital and *Enterobacteriaceae* species.

## 3. Discussion

The current study is the first report showing the molecular epidemiology of ESBL producing *Enterobacteriaceae* among patients investigated for sepsis at four referral hospitals located in the northern, central and southern parts of Ethiopia. Of all the *Enterobacteriaceae* subjected to WGS, 75.5% encoded at least one ESBL gene. Similar to our study, the previous reports from different African countries also showed a high occurrence of ESBL [24,25,26]. The high frequency of *Enterobacteriaceae* producing ESBL enzymes among sepsis patients in Ethiopia also showed similarities with studies conducted in other continents [4,27,28,29]. The spread of bacteria producing ESBL enzymes among sepsis patients is worrying because of the invasive nature of the disease, coupled with the limited antibiotics options left for its management. More importantly, the high level of ESBL producing strains among sepsis causing *Enterobacteriaceae* in low-income countries is a major public health problem, due to the limited laboratory services and therapeutic options available. ESBL are often encoded by plasmids that are able to transfer horizontally, which contributes to the rapid spread of ESBL-pE. Additionally, as documented for developed countries, the ESBL genes detected in food, animals and environment, human migration and limited access to sanitations could also contribute to the rise of ESBL genes globally [30]; however, this needs further investigation for the case of Ethiopia.

In a comparison between the hospitals, very high ESBL-pE levels were detected in the three hospitals, HUCSH (95%), Y12HMC (90%) and TASH (82%), which are located in the southern and central parts of Ethiopia. In contrast, the detection of ESBL enzymes at DRH (55.8%), which is located in the northern part of Ethiopia, was comparatively low. However, the lower level of ESBL detection at DRH was still higher compared to studies conducted in South Africa [31], Mozambique [20] and South and South East Asia [32]. While it is known that the spread of ESBL genes varies geographically [33], the factors that contribute to its high occurrence in some hospitals and a lower detection rate in the other hospital within a single country requires investigation so that effective control mechanisms can be designed.

In this study, *bla*_CTX-M_ (73%) was the most frequently detected ESBL family across the four referral hospitals. This finding was similar to a multicenter study performed in neighboring Sudan that reported 78% of *bla*_CTX-M_ [25]; however, a very low detection rate was reported in the U.S.A. [34]. *Bla*_CTX-M-15_ (70.4%) was the most abundant ESBL gene detected. This finding showed similarities with different studies across the globe [1,14,20,27,29,32]. *Bla*_CTX-M-15_ was very abundant at TASH (73.6%) and HUCSH (93%), while it was detected at a comparatively lower rate at DRH (50.4%). While *bla*_CTX-M-15_ was disseminated in all hospitals, *bla*_CTX-M-3_ and *bla*_CTX-M-9_ were detected only at DRH (northern). The detection of high levels of *bla*_CTX-M_ at TASH (central) may possibly be explained because the hospital is the main destination of patients referred from all over the country, and these patients can carry such strains to the hospital.

In this study, *bla*_TEM_ (73%) was also detected in abundance, which showed agreements with other studies [12,33]. Out of these, only *bla*_TEM-207_ were recorded as an ESBL gene, though its detection rate was rare and restricted to only two hospitals (TASH and DRH). This rare detection of *bla*_TEM_ ESBL variants showed similarities with a study conducted in Mozambique that reported a single detection of the *bla*_TEM_ gene [20]. Of several variants of *bla*_TEM_ detected, *bla*_TEM-1B_ (61.5%) was the most frequent. Other variants of *bla*_TEM_ were either broad-spectrum beta-lactamases or inhibitor-resistant beta-lactamases. Even though most *bla*_TEM_ beta-lactamases were not ESBL, it is worrying that the majority of *bla*_TEM_ variants were co-detected with ESBL genes, such as *bla*_CTX-M_ variants and other ESBL genes.

Similarly, the detection of *bla*_SHV_ (33%) showed similarities with a study conducted in Sudan that reported 28% of the *bla*_SHV_ genes [25]. The ESBL variants of *bla*_SHV_ detected in this study were *bla*_SHV-12_, *bla*_SHV-65_ and *bla*_SHV-106_. The *bla*_SHV-106_ variant was detected at DRH (northern) and TASH (central), but not at Y12HMC. A similar *bla*_SHV-106_ detection rate was reported in Portugal [35]. However, a very minimal *bla*_SHV_ occurrence was reported in China that investigated 499 E.coli [36]. *bla*_SHV-187_ (27.6%), was the most frequently detected variant of *bla*_SHV_ and is a broad-spectrum beta-lactamase. A majority of the *bla*_SHV_ genes were co-detected with *bla*_CTX-M_ and other ESBL genes, which worsens the problem.

In addition, different intrinsic *bla*_OXY_ variants encoded with K. oxytoca that are ESBL were detected even though most were found at TASH and DRH, but rarely at HUCSH and Y12HMC. After the first detection of *bla*_OXY_ in Spain in 2009 [37], several variants were detected in this study showing how fast the variants are spreading throughout the world [38,39]. In addition to *bla*_GES-11_, the rarely detected ESBL genes that are grouped in class C and has having ESBL activities were *bla*_SRT-1_ and *bla*_CMY-42_, and this showed agreements with other studies [40,41].

Multiple ESBL genes were detected in several cases and most ESBL-encoding strains were harboring other beta-lactamase genes. The *bla*_CTX-M-15_ gene co-occurred with several other ESBL genes and the non-ESBL variants of *bla*_TEM_ and *bla*_SHV_. The co-occurrence of *bla*_CTX-M-15_ and *bla*_TEM-1B_ was the most frequently detected gene combination, followed by the *bla*_CTX-M_ and *bla*_SHV-187_ combination. The findings of this co-occurrence of multiple ESBL genes were in agreement with the other studies [20,25]. These findings showed the successful spread of *Enterobacteriaceae* harboring multiple ESBL genes in the study settings, which is very alarming.

In the current study, 14% of *Enterobacteriaceae* harbored at least one AmpC gene while seven strains had multiple AmpC genes. However, a higher AmpC detection was reported in Cairo, Egypt [16]. Several variants of AmpC were detected, with *bla*_ACT_ (*n* = 25) as the most common variant, which was in accordance with another study [19]. *bla*_ACT-5_, *bla*_ACT-7_, *bla*_ACT-16_, *bla*_ACT-14_ and *bla*_ACT-15_ were the different variants of *bla*_ACT_ detected in this study. Another commonly detected AmpC gene was *bla*_CMY_ and its different variants *bla*_CMY-6_, *bla*_CMY-148_, *bla*_CMY-2_ and *bla*_CMY-42_. The detection of *bla*_CMY_ was in agreement with the other studies [19,20]. The other rarely detected AmpC genes detected were *bla*_DHA-1_, *bla*_CMH-3_, *bla*_SRT-1_, *bla*_SRT-2_ and *bla*_ADC-25_. A majority of AmpC producing *Enterobacteriaceae* (71%) were also ESBL producers that could easily facilitate the rapid spread of both ESBL and AmpC genes, as described in a review article from Africa [24].

At least one ESBL gene was detected among 95% of *K. pneumoniae*, 68% of *K. variicola*, 53% *E. coli* and 43% *of E. cloacae*. These findings were in line with other studies that recognized these organisms as the main ESBL carriers [9,10,42]. *K. pneumoniae* was the most frequent isolate that harbored high frequencies of ESBL *bla*_CTX-M-15_ (92%), and also showed high frequencies of *bla*_SHV-187_ (78%) and *bla*_TEM-1B_ (74%). A similar high level of ESBL genes encoded by *K. pneumoniae* were reported in Tanzania [43]; however, a very low detection rate was reported in Germany [10] and the U.S.A. [34]. In addition to the ESBL genes, 9% of *K. pneumoniae* were harboring different AmpC genes showing that these strains are the main ESBL carriers in all hospitals. The detection of ESBL genes in sepsis isolates is very important to guarantee that appropriate antibiotic therapy is prescribed when an ESBL producing strain is identified, and to limit unnecessary antibiotic prescriptions when ESBL genes are not identified [34].

## 4. Materials and Methods

### 4.1. Study Design and Study Sites

**Figure 5:**
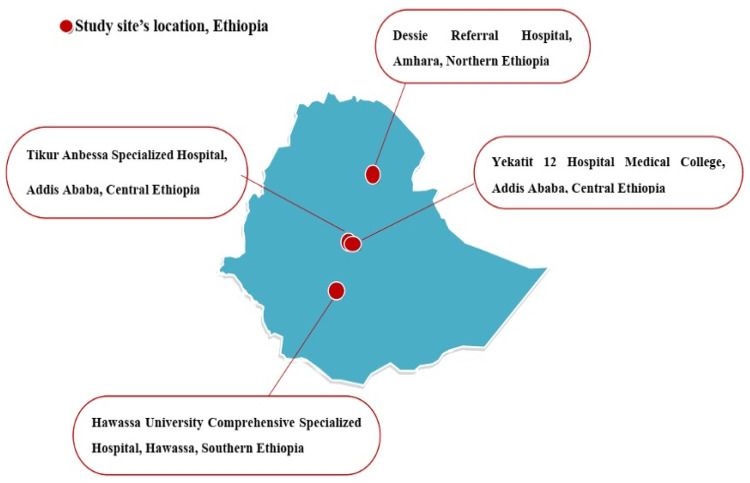
The four Ethiopian referral hospitals selected for this study and from where *Enterobacteriaceae* were isolated. TASH, located in Addis Ababa, is the teaching hospital of Addis Ababa University and the largest referral and oldest hospital in the country. It provides tertiary level referral diagnoses and treatment for patients referred from all over the country with over 800 beds. DRH is one of the largest public hospitals in the northern part of Ethiopia, located in Dessie. It is a referral hospital with 560 beds, providing services for the surrounding areas and residents of the neighboring regions. Y12HMC is located in Addis Ababa and provides health care services to Addis Ababa residents, referral cases from health centers in Addis Ababa and its bordering regions. The hospital has over 300 beds and serves more than 5 million people in its catchment area. HUCSH, located in Hawassa city, is one of the largest health facilities in the southern part of the country and provides teaching, public health services and research activities with over 400 beds.

Among the patients investigated for sepsis, a multicenter prospective cross-sectional study was conducted between October 2019 and September 2020 at four selected hospitals located in the central, southern and northern parts of Ethiopia (Figure 5). University and referral hospitals that had established microbiology laboratories or a link with a nearby government regional microbiology laboratory were selected. These were the Tikur Anbessa Specialized Hospital (TASH) and the Yekatit 12 Specialized Hospital Medical College (Y12HMC) in the central, Hawassa University Comprehensive Specialized Hospital (HUCSH) in the southern and Dessie Referral Hospital (DRH) in the northern parts of Ethiopia.

### 4.2. Blood Culture and Enterobacteriaceae Isolation and Identification

All patients investigated for sepsis who sought medical service at the study sites were included. The attending physician’s decision was used to identify the eligible patients as sepsis cases and their socio-demographic and possible risk factors data were gathered. All age groups were included, but patients who had been on antibiotic treatment within the preceding ten days were excluded from the study. From all the study sites, a total of 1416 clinically diagnosed cases of sepsis from different wards were enrolled in the study. A single blood culture bottle system was processed from all the patients, and bacterial identification was performed in accordance with a standardized laboratory protocol. At each study site, *Enterobacteriaceae* were characterized by their colony characteristics, Gram-staining and conventional biochemical tests using triple sugar iron, indole, urea, citrate, lysine decarboxylase, motility and malonate. All strains were stored at −70 °C or −16 °C and transported to the Armauer Hansen Research Institute, and later brought to Sweden for further characterization. All the *Enterobacteriaceae* were re-identified and confirmed using MALDI-TOF MS at the Clinical Microbiology Department of Uppsala University Hospital, Uppsala, Sweden, and the Karolinska Institute, Stockholm, Sweden. Each laboratory test was processed in accordance with the established protocols and carefully recorded. Each MALDI-TOF run included quality control using *E. coli* ATCC 25922. A total of 301 *Enterobacteriaceae* isolated from all the study sites was subjected for whole genome sequencing (WGS) for the current analysis. The number of *Enterobacteriaceae* subjected to WGS from DRH, TASH, HUCSH and Y12HMC were 113, 91, 57 and 40, respectively.

### 4.3. DNA Extraction, Whole Genome Sequencing (WGS) and the Identification of Resistance Genes

From all the Enterobacteriaceae, DNA was extracted manually using a QIAamp DNA Mini Kit (QIAGEN, Hilden, Germany), according to the manufacturer’s instructions. DNA extractions were performed by taking 2–5 pure colonies that grew on cystine lactose electrolyte deficient agar at 37 °C for 24 h aerobically. After the extraction, the DNA concentrations were measured with QubitTM3.0 (Thermo scientific, Waltham, MA, USA). All the extracted DNA samples were kept at −20 °C until they were submitted for whole genome sequence determination.

All the *Enterobacteriaceae* were subjected to WGS at the Science for Life Laboratory, Solna, Sweden. From each DNA sample, 20 μL was transferred into a 96-well WGS plate. Sequencing libraries were generated using Nextera XT (Illumina kits) and short-read sequencing was run on Illumina (HiSeq 2500) systems with a 150 bp insert size paired end sequencing protocol at the Science for Life Laboratory. SPAdes (version 3.9) was used for the genome assembly. With the assembled genomes, the acquired antimicrobial resistance genes were identified using the ResFinder 4.1 web tool at the Center for Genomic Epidemiology http://www.genomicepidemiology.org/ (accessed on 16 August 2021) using a threshold of 90% and 60% coverage. Each WGS run included quality control.

### 4.4. Statistical Analysis

The data was prepared using a Microsoft Office Excel sheet and was imported to SPSS version 27 for analysis. The frequencies of the resistance genes, ESBL and AmpC producers and the co-occurrence of multiple ESBL genes and other variables were calculated. The binary logistic regression analysis was used to observe the associations of the sociodemographic and possible risk factors of ESBL producing Enterobacteriaceae. A *p*-value < 0.05 was considered as statistically significant.

## 5. Conclusions

To our knowledge, this is the first multicenter study that reported the molecular epidemiology of ESBL and AmpC producing *Enterobacteriaceae* among sepsis patients at four Ethiopian hospitals located in the northern, central and southern parts of the country. A very high genotypic frequency of ESBL producing *Enterobacteriaceae* among sepsis patients was detected. Several variants of ESBL genes were detected and the most frequent ESBL gene was *bla*_CTX-M-15_. In addition to the ESBL genes, diverse variants of *bla*_TEM_ and *bla*_SHV_ beta-lactamases were detected, where *bla*_TEM-1B_ and *bla*_SHV-187_ were the most frequently detected variants in their respective families. Multiple combinations of ESBL genes were detected and most ESBL genes were concurrently detected with the *bla*_TEM-1B_ and *bla*_SHV-187_ beta-lactamases. Different AmpC genes were detected in some *Enterobacteriaceae* where variants of *bla*_ACT_ and *bla*_CMY_ were commonly detected. The current findings strongly suggest the urgent need for high standard bacteriological laboratory services to guide the antibiotic treatment of sepsis and other life-threatening infections due to ESBL and AmpC producing Enterobacteriaceae. It is also helpful for effective antimicrobial stewardship so that infection control programs can be improved.

## Figures and Tables

**Table 1:** Sociodemographic data of the patients investigated for sepsis at four different hospitals in Ethiopia.

Sociodemographic Data	Number of Patients		Percentage
Hospitals	TASH	501	35.4
	Y12HMC	298	21
	DRH	301	21.3
	HUCSH	316	22.3
Gender	Male	783	55.3
	Female	633	44.7
Age category	<29 days	586	41.4
	≥30 days to ≤1 year	256	18.1
	1–5 years	135	9.5
	5–18 years	158	11.2
	≥18 years	281	19.8
Ward	EOPD	104	7.3
	ICU	38	2.7
	Medical ward	148	10.5
	NICU	596	42.1
	Pediatrics	497	35.1
	Surgical ward	33	2.3
Referral patient	Yes	722	51
	No	694	49
Previous admission	Yes	299	21.1
	No	1117	78.9
Hospital stay duration	1 week	828	58.5
	2 weeks	222	15.7
	3 weeks	146	10.3
	4 weeks and above	220	15.5
Underlying diseases	Yes	665	47
	No	751	53
Previous antibiotic treatment before recruitment to the study	Yes	440	31.1
	No	976	68.9
Fever	Up to 3 days	687	64.4
	4–6 days	207	19.4
	7 days and above	173	16.2
	No	349	24.6
	Total	1416	100

TASH—Tikur Anbessa Specialized Hospital; Y12HMC—Yekatit 12 Specialized Hospital Medical College; DRH—Dessie Referral Hospital; HUCSH—Hawassa University Comprehensive Specialized Hospital; EOPD—emergency outpatient department; ICU—intensive care unit and NICU—neonatal intensive care unit.

**Table 2:** Frequency and distribution of *Enterobacteriaceae* isolated from the patients investigated for sepsis and subjected for whole genome sequence in four Ethiopian hospitals.

*Enterobacteriaceae*	DRH *n* (%)	TASH *n* (%)	HUCSH *n* (%)	Y12HMC *n* (%)
*Klebsiella pneumoniae* (*n* = 103)	12(11)	39(43)	22(39)	30(75)
*Klebsiella variicola* (*n* = 74)	44(39)	2(2)	28(49)	-
*Escherichia coli* (*n* = 53)	17(15)	28(31)	4(7)	4(10)
*Enterobacter cloacae* (*n* = 21)	10(9)	6(7)	1(2)	4(10)
*Pantoea dispersa* (*n* = 20)	20(18)	-	-	-
*Klebsiella oxytoca* (*n* = 13)	5(4)	6(7)	1(2)	1(3)
*Enterobacter xiangfangensis* (*n* = 3)	3(3)	-	-	-
*Raoultella ornithinolytica* (*n* = 2)	1(1)	1(1)	-	-
*Serratia marcescens* (*n* = 2)	-	2(2)	-	-
*Leclercia adecarboxylata* (*n* = 2)	-	2(2)	-	-
*Achromobacter xylosoxidans* (*n* = 1)	-	-	1(2)	-
*Enterobacter bugandensis* (*n* = 1)	-	1(1)	-	-
*Enterobacter kobei* (*n* = 1)	-	1(1)	-	-
*Enterobacter ludwigii* (*n* = 1)	-	1(1)	-	-
*Kosakonia cowanii* (*n* = 1)	1(1)	-	-	-
*Lelliottia amnigena* (*n* = 1)	-	1(1)	-	-
*Salmonella* spp. (*n* = 1)	-	-	-	1(3)
*Shigella dysenteriae* (*n* = 1)	-	1(1)	-	-
Total (*n* = 301)	113	91	57	40

TASH—Tikur Anbessa Specialized Hospital; Y12HMC—Yekatit 12 Specialized Hospital Medical College; DRH—Dessie Referral Hospital and HUCSH—Hawassa University Comprehensive Specialized Hospital.

**Table 3:** Frequency of *Enterobacteriaceae* that harbored at least one ESBL gene in relation to patient characteristics.

Patient Characteristics		ESBL		Odds Ratio			
		Producer *n* (%)	Non-Producer *n* (%)	COR (95% CL)	*p*-Value	AOR (95%CL)	*p*-Value
Hospital	DRH (*n* = 113)	63(55.8)	50(44.2)	0.140(0.047–0.420)	0.001	0.111(0.031–0.403)	0.001
	TASH (*n* = 91)	75(82)	16(18)	0.521(0.162–1.671)	0.273		
	HUCSH (*n* = 57)	54(95)	3(5)	0.200(0.422–9.472)	0.382		
	Y12HMC (*n* = 40)	36(90)	4(10)	Constant			
Gender	Male (*n* = 174)	128(73.6)	46(26.4)	0.751(0.437–1.292)	0.301		
	Female (*n* = 127)	100(78.7)	27(21.3)	Constant			
Age category	≤29 days (*n* = 187)	137(73)	50(27)	1.534(0.739–3.184)	0.250		
	30 days–≤1 year (*n* = 32)	30(94)	2(6)	8.4(1.741–40.529)	0.008	4.564(0.285–73.109)	
	>1–≤5 year (*n* = 21)	18(86)	3(14)	3.360(0.840–13.441)	0.087		
	>5–<18 year (*n* = 22)	18(82)	4(18)	2.520(0.711–8.934)	0.152		
	≥18 years (*n* = 39)	25(64)	14(36)	Constant			
Ward	EOPD (*n* = 12)	9(75)	3(25)	3.750(0.587–23.936)	0.162		
	ICU (*n* = 8)	5(63)	3(38)	2.083(0.298–14.549)	0.459		
	Medical ward (*n* = 15)	12(80)	3(20)	5.000(0.806–13.457)	0.084		
	NICU (*n* = 189)	139(73.5)	50(26.5)	3.475(0.897–13.457)	0.071		
	Paediatrics (*n* = 68)	59(87)	9(13)	8.194(1.846–36.366)	0.006	3.722(0.209–66.238)	0.371
	Surgical ward (*n* = 9)	4(44)	5(56)	Constant			
Hospital stay duration	1 week (*n* = 194)	135(69.6)	30.4)	0.277(0.094–0.818)	0.020	0.268(0.069–1.037)	0.056
	2 weeks (*n* = 37)	33(89.2)	4(10.8)	0.808(0.164–3.989)	0.794		
	3 weeks (*n* = 23)	20(87)	3(13)	Constant			
	≥4 weeks (*n* = 47)	40(85.1)	7(14.9)	0.693(0.186–2.572)	0.583		
Underlying diseases	Yes (*n* = 117)	99(84.6)	18(15.4)	2.363(1.306–4.278)	0.004	1.197(0.308–4.652)	0.795
	No (*n* = 184)	129(70.1)	55(29.9)	Constant			
Previous hospitalization	Yes (48)	38(79.2)	10(20.8)	1.267(0.597–2.689)	0.538		
	No (*n* = 253)	190(75.1)	63(24.9)	Constant			
Referred patient	Yes (*n* = 140)	109(77.9)	31(22.1)				
	No (*n* = 161)	119(73.9)	42(26.1)	1.252(0.735–2.131)	0.409		
Previous antibiotic treatment history	Yes (*n* = 52)	47(90.4)	5(9.4)	0.283(0.108–0.742)	0.01	0.639(0.179–2.281)	0.490
	No (*n* = 249)	181(72.7)	68(27.3)	Constant			

TASH—Tikur Anbessa Specialized Hospital; Y12HMC—Yekatit 12 Specialized Hospital Medical College; DRH—Dessie Referral Hospital and HUCSH—Hawassa University Comprehensive Specialized Hospital.

**Table 4:** Frequency and distribution of ESBL-encoding genes detected at four Ethiopian hospitals.

ESBL Genes	Total	Hospitals				ESBL Gene Harboring *Enterobacteriaceae*											
		DRH *n* (%)	HUCSH *n* (%)	TASH *n* (%)	Y12HMC *n* (%)	*K. pneumoniae* *n* (%)	*K. variicola* *n* (%)	*E. coli* *n* (%)	*E. cloacae* *n* (%)	*P. dispersa* *n* (%)	*K. oxytoca* *n* (%)	*E. xiangfangensis* *n* (%)	*S. marcescens* *n* (%)	*R. ornithinolytica* *n* (%)	*L. adecarboxylata* *n* (%)	*Salmonella* spp. *n* (%)	*E. kobei* *n* (%)
*bla*_CTX-M-15_	212(70.4) *	57(50.4)	53(93)	67(73.6)	35(87.5)	95(92)	48(65)	27(51)	8(38)	18(90)	9(69)	2(67)	1(50)	1(50)	1(50)	1(100)	1(100)
*bla*_CTX-M-3_	3(1)	3(2.7)	-	-	-	-	2(3)	-	-	-	-	-	-	1(50)	-	-	-
*bla*_CTX-M-9_	1(0.3)	1(0.9)	-	-	-	-	-	-	-	1(5)	-	-	-	-	-	-	-
*bla*_SHV-106_	11(3.7)	5(4.4)	1(1.8)	5(5.5)	-	11(11)	-	-	-	-	-	-	-	-	-	-	-
*bla*_SHV-65_	2(0.7)	-	-	-	2(5)	2(2)	-	-	-	-	-	-	-	-	-	-	-
*bla*_SHV-12_	1(0.3)	-	-	1(1.1)	-	-	-	1(2)	-	-	-	-	-	-	-	-	-
*bla*_TEM-207_	3(1)	1(0.9)	-	2(2.2)	-	2(2)	-	-	-	1(5)	-	-	-	-	-	-	-
*bla*_OXY-1-2_	4(1.3)	(1.8)	1(1.8)	1(1.1)	-	-	-	-	-	-	4(31)	-	-	-	-	-	-
*bla*_OXY-1-5_	3(1)	-	-	2(2.2)	1(2.5)	-	-	-	-	-	2(15)	-	-	1(50)	-	-	-
*bla*_OXY-1-3_	2(0.7)	1(0.9)	-	1(1.1)	-	-	-	-	-	-	2(15)	-	-	-	-	-	-
*bla*_OXY-2-2_	1(0.3)	-	-	1(1.1)	-	-	-	-	-	-	1(8)	-	-	-	-	-	-
*bla*_OXY-2-7_	1(0.3)	-	-	1(1.1)	-	-	-	-	-	-	1(8)	-	-	-	-	-	-
*bla*_OXY-2-8_	1(0.3)	1(0.9)	-	-	-	-	-	-	-	-	1(8)	-	-	-	-	-	-
*bla*_OXY-4-1_	1(0.3)	1(0.9)	-	-	-	-	-	-	-	-	1(8)	-	-	-	-	-	-
*bla*_OXY-5-1_	1(0.3)	-	-	1(1.1)	-	-	-	-	-	-	1(8)	-	-	-	-	-	-
*bla*_SRT-1_	2(0.7)	-	-	2(2.2)	-	-	-	-	-	-	-	-	2(100)	-	-	-	-
*bla*_CMY-42_	1(0.3)	-	-	1(1.1)	-	-	-	1(2)	-	-	-	-	-	-	-	-	-
*bla*_GES-11_	1(0.3)	-	-	-	1(2.5)	-	-	-	1(5)	-	-	-	-	-	-	-	-

TASH—Tikur Anbessa Specialized Hospital; Y12HMC—Yekatit 12 Specialized Hospital Medical College; DRH—Dessie Referral Hospital and HUCSH—Hawassa University Comprehensive Specialized Hospital. *—frequently detected.

**Table 5:** Frequency and distribution of other *bla*_TEM_ and *bla*_S__HV_ variants detected at four Ethiopian hospitals.

	Total *n*(%)		Total Detection per Study Site			
			DRH *n* (%)	HUCSH *n* (%)	TASH *n* (%)	Y12HMC *n* (%)
TEM Variants Detected	*bla*_TEM-1B_	185(61.5) *	65(57.5)	46(80.7)	45(49.5)	29(72.5)
	*bla*_TEM-1A_	5(0.9)	1(0.9)	1(20)	2(2.2)	1(2.5)
	*bla*_TEM-1C_	1(0.3)	1(0.9)	-	-	-
	*bla*_TEM-206_	2(0.7)	1(0.9)	-	1(1.1)	-
	*bla*_TEM-219_	2(0.7)	-	1(1.8)	1(1.1)	-
	*bla*_TEM-30_	3(1)	1(0.9)	-	2(2.2)	-
SHV variants detected	*bla*_SHV-187_	187(27.6) *	11(9.7)	20(35.1)	38(41.8)	14(35)
	*bla*_SHV-28_	10(3.3)	4(3.5)	1(1.8)	5(5.5)	-
	*bla*_SHV-79_	8(2.7)	-	-	-	8(20)
	*bla*_SHV-85_	8(2.7)	-	-	-	8(20)
	*bla*_SHV-89_	8(2.7)	-	-	-	8(20)
	*bla*_SHV-40_	7(2.3)	-	-	-	7(17.5)
	*bla*_SHV-60_	5(1.7)	-	-	-	5(12.5)
	*bla*_SHV-119_	2(0.7)	-	-	2(2.2)	-
	*bla*_SHV-94_	1(0.3)	-	-	-	1(2.5)
	*bla*_SHV-172_	1(0.3)	-	-	-	1(2.5)
	*bla*_SHV-56_	8(2.7)	-	-	-	8(20)

TASH—Tikur Anbessa Specialized Hospital; Y12HMC—Yekatit 12 Specialized Hospital Medical College; DRH—Dessie Referral Hospital and HUCSH—Hawassa University Comprehensive Specialized Hospital. *—frequently detected.

**Table 6:** Co-occurrence of multiple ESBL genes and ESBL genes with other common non-ESBL variants of *bla*_TEM_ and *bla*_SHV_.

Combinations of Genes	Total *n*	DRH *n*	HUCSH *n*	TASH *n*	Y12HMC *n*
*bla*_CTX-M-15_ * *bla*_TEM-1B_	166	53	45	39	29
*bla*_CTX-M-15_ * *bla*_SHV-187_	78	11	20	34	13
*bla*_CTX-M-15_ * *bla*_SHV-106_	10	5	1	4	-
*bla*_CTX-M-15_ * *bla*_OXY-1-2_	3	2	1	-	-
*bla*_CTX-M-15_ * *bla*_TEM-207_	1	-	-	1	-
*bla*_CTX-M-15_ * *bla*_OXY-1-5_	3	-	-	2	1
*bla*_TEM-1B_ * *bla*_SHV-106_	10	5	-	5	-
*bla*_TEM-1B_ * *bla*_OXY-1-2_	2	2	-	-	-
*bla*_TEM-1B_ * *bla*_OXY-1-5_	3	-	-	2	1
*bla*_TEM-1B_ * *bla*_TEM-207_	3	1	0	2	1
*bla*_SHV-187_ * *bla*_SHV-106_	11	5	1	5	-
*bla*_SHV-187_ * *bla*_TEM-207_	2	-	-	2	-
*bla*_SHV-187_ * *bla*_TEM-207_	2	-	-	2	-
*bla*_CTX-M-15_ * *bla*_TEM-1B_ * *bla*_SHV-187_	56	9	15	24	8
*bla*_CTX-M-15_ * *bla*_TEM-1B_ * *bla*_SHV-106_	9	5	-	4	-
*bla*_CTX-M-15_ * *bla*_TEM-1B_ * *bla*_OXY-1-5_	3	-	-	2	-
*bla*_CTX-M-15_ * *bla*_TEM-1B_ * *bla*_TEM-207_	1	-	-	1	-
*bla*_CTX-M-15_ * *bla*_SHV-187_ * *bla*_SHV-106_	10	5	1	4	-
*bla*_CTX-M-15_ * *bla*_SHV-106_ * *bla*_TEM-207_	1	-	-	1	-
*bla*_SHV-106_ * *bla*_TEM-1B_ * *bla*_SHV-187_	10	5	-	5	-
*bla*_TEM-207_ * *bla*_TEM-1B_ * *bla*_SHV-187_	2	-	-	2	-
*bla*_CTX-M-15_ * *bla*_TEM-1B_ * *bla*_SHV-187_ * *bla*_SHV-106_	9	5	-	4	-
*bla*_CTX-M-15_ * *bla*_TEM-1B_ * *bla*_SHV-187_ * *bla*_TEM-207_	1	-	-	1	-
*bla*_TEM-207_ * *bla*_TEM-1B_ * *bla*_SHV-187_ * *bla*_SHV-106_	2	-	-	2	-
*bla*_CTX-M-15_ * *bla*_TEM-1B_ * *bla*_SHV-187_ * *bla*_TEM-207_ * *bla*_SHV-106_	1	-	-	1	-

“*” means “and”.

**Table 7:** Frequency and distribution of the AmpC genes detected at four Ethiopian hospitals.

AmpC Genes	Hospital				AmpC Harboring *Enterobacteriaceae*								
	DRH *n*	HUCSH *n*	TASH *n*	Y12HMC *n*	*K. pneumoniae* *n*	*K. variicola* *n*	*E. coli* *n*	*E.cloacae* *n*	*K. oxytoca* *n*	*E. xiangfangensis* *n*	*S. marcescens* *n*	*E. ludwigii* *n*	*E. kobei* *n*
*bla*_ACT-__7_ (*n* = 9)	5	-	1	3	-	-	-	6	-	2	-	-	1
*bla*_CMY-6_ (*n* = 7)	2	-	5	-	7	-	-	-	-	-	-	-	-
*bla*_ACT-__16_ (*n* = 6)	1	-	5	-	-	-	-	6	-	-	-	-	-
*bla*_ACT-__14_ (*n* = 3)	3	-	1	-	-	-	-	4	-	-	-	-	-
*bla*_ACT-__5_ (*n* = 2)	3	-	-	-	-	-	-	3	-	-	-	-	-
*bla*_DHA-1_ (*n* = 2)	-	5	-	-	2	2	-	-	1	-	-	-	-
*bla*_CMY-__148_ (*n* = 3)	-	1	2	-	-	-	2	-	-	-	-	1	-
*bla*_CMY-__2_ (*n* = 2)	-	-	2	-	-	-	2	-	-	-	-	-	-
*bla*_CMH-3_ (*n* = 2)	2	-	-	-	-	-	-	2	-	-	-	-	-
*bla*_SRT-1_ (*n* = 2)	-	-	2	-	-	-	-	-	-	-	2	-	-
*bla*_ACT-15_ (*n* = 1)	-	1	-	-	-	-	-	1	-	-	-	-	-
*bla*_ACT-4_ (*n* = 1)	1												
*bla*_CMY-42_ (*n* = 1)	-	-	1	-	-	-	1	-	-	-	-	-	-
*bla*_SRT-2_ (*n* = 1)	-	-	1	-	-	-	-	-	-	-	1	-	-
Total AmpC genes detected	17	7	20	3	9	2	5	22	1	2	1	1	1

## Data Availability

The data presented in this study are available on request from the corresponding author. The data are not publicly available due to privacy restrictions.
